# Evaluation of the cytotoxicity, apoptotic effects, and remineralization potential of recent bioceramic-based root canal sealers

**DOI:** 10.1016/j.jobcr.2025.05.002

**Published:** 2025-05-10

**Authors:** Manar M. Galal, Amira Galal Ismail, Yousra Nashaat, Tamer M. Hamdy

**Affiliations:** a Restorative and Dental Materials Department, Oral and Dental Research Institute, National Research Centre (NRC), Giza, Dokki, 12622, Egypt; b Endodontic Department, Faculty of Dentistry, October University, Cairo, Egypt

**Keywords:** Bioceramic sealer, VDW-1Seal, Fill root ST, ADseal, Apoptosis, Cytotoxicity, Flow cytometry, MTT, Remineralization

## Abstract

**Background:**

This study is aimed at evaluating the cytotoxicity, apoptosis/necrosis, and calcium ion release of ADseal (a resin-based sealer) and two recently introduced bioceramic-based root canal sealers, which are VDW.1 seal bioceramic and Fill Root ST.

**Methods:**

The cellular responses were studied in human periodontal ligament stem cells (hPDLSCs). Four root canal sealer extracts concentrations (100 %, 50 %, 25 %, and 12.5 %) were assessed for cell viability using the cell viability MTT essay and after 24 h, 48 h, and 7 days for all concentrations. To determine the effect of materials on apoptosis and necrosis, flow cytometry analysis was used. Calcium ion release was evaluated after 14 days of storage in distilled water using optical emission spectroscopy (ICP-OES). A two-way ANOVA was used for the statistical analysis of the acquired results.

**Results:**

Fill Root ST and VDW.1 seal bioceramic showed the highest amount of cell viability compared to ADseal sealer. Moreover, Fill Root ST, followed by VDW.1 seal bioceramic, demonstrated decreased cell apoptosis and necrosis. Moreover, VDW.1 seal bioceramic showed the highest calcium ion release, followed by Fill Root ST. ADseal sealers showed the lowest calcium ion release compared to bioceramic-based root canal sealers.

**Conclusions:**

Based on the results of this study, in terms of cytotoxicity, apoptosis/necrosis, and remineralization ability, bioceramic-based root canal sealers were more biocompatible than ADseal resin-based sealers.

## Highlights

• Fill Root ST, VDW.1 seal, and ADseal sealer.
• Bioceramic-based root canal sealers, and resin-based sealer.
• Cytotoxicity, apoptosis/necrosis, and calcium ion release.

## Graphical abstract

**Figure:**
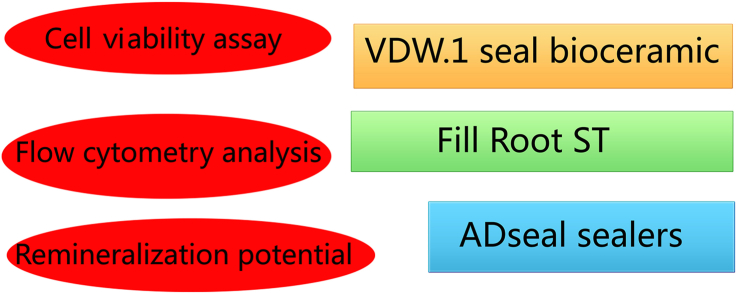

## 1 Background

Getting rid of root canal infections and creating the ideal root canal cleaning and shaping in order to prevent more infections and apical periodontitis are the primary goals of root canal therapy.^1^ The creation of appropriate endodontic therapy requires continuous advancements in both root canal instruments and root canal materials.^2^, ^3^, ^4^, ^5^ There is a wide variety of root canal sealers on the market; each has unique qualities. Currently, the most often utilized root canal sealer materials are resin-based sealers, which offer sufficient physical qualities and a satisfactory apical closure.^6^^,^^7^ There is a lack of bioactivity and remineralization capability between the root canal sealer and the tooth structure.^8^^,^^9^ As a result, novel bioactive materials have emerged and are being used in dentistry for a range of clinical purposes, such as apexification, vital pulp therapy, root canal filling, and perforation repair.^9^, ^10^, ^11^, ^12^, ^13^, ^14^ These bioactive materials, when in contact with dental tissues, help in the process of remineralization.^15^

Bioceramic-based sealers have been widely used in endodontics due to their superior biological and physicochemical characteristics.^16^^,^^17^ These materials consist of tricalcium aluminate or dicalcium/tricalcium silicates. They can produce hydroxyapatite on their surface when in contact with tissue fluids; therefore, bioceramic-based sealers are considered a bioactive material.^9^^,^^18^, ^19^, ^20^

VDW-1 seal A novel premixed bioceramic sealer has been developed for use as root canal and permanent filler materials. The main components of the single-syringe VDW-1 sealer are zirconium dioxide and calcium aluminosilicate. The bioactivity of VDW-1 sealer is attributed by the manufacturer to the release of calcium ions, which in turn encourage the creation of mineralized tissue. Fill Root ST is a recently introduced calcium silicate bioceramic-based sealer. It is claimed to have enhanced physical and biological properties and be able to release calcium ions. Moreover, the manufacturer claims its favourable handling characteristics and its ability to induce mineralized tissue formation by increasing alkalinity and releasing calcium ions. Nevertheless, so far, not enough research has been done to assess its impact on periapical tissues and associated cells.

Cytotoxicity, biocompatibility, and bioactive potentiality all contribute to the explanation of the biological interaction between the surrounding tissues and the root canal sealers.^21^ The capacity of a chemical to change a cell's viability is known as cytotoxicity, and it can be assessed at several physiological parameters, including reduced cell division and growth, necrosis, apoptosis, or combinations of these characteristics. Since the potential intracanal sealer cytotoxic activity has an impact on the physiological and biological behavior of these cells, it is crucial to evaluate it. Till now, there is not enough data available about the cytotoxicity, apoptotic effects, and release of calcium ions with the consequence of the reminerlization potential of two recent bioceramic-based root canal sealers (VDW-1 seal bioceramic and Fill Root ST). Therefore, this laboratory study's objective was to compare the cytotoxicity, apoptotic effects, and release of calcium ions of two recently introduced bioceramic-based root canal sealers (Fill Root ST and VDW-1 seal bioceramic) to an epoxy resin-based root canal sealer (ADseal root canal sealer). The null hypothesis proposed that, as regards cytotoxicity, apoptotic effects, and calcium ion release, there was no difference between resin-based root canal sealers (ADseal root canal sealers) and the novel bioceramic-based sealers (VDW.1 seal bioceramic and Fill Root ST).

## 2 Methods

The study was approved by the Medical Research Ethical Committee (MREC) of the National Research Centre (NRC), Cairo, Egypt (approval number for the study: 36572022). The root canal sealers examined in the current study are shown in Table 1.

**Table 1:** The root canal sealers utilized in the study and their chemical composition.

Root canal sealers	Chemical composition^a^	Manufacturers
VDW.1 seal bioceramic	Zirconium dioxide (50–70 %), tricalcium silicate (5–15 %), dimethyl sulfoxide (10–30 %), lithium carbonate (<0.5 %), thickening agents (<6 %).	*VDW, München, Germany*
Fill Root ST	Zirconium oxide, dicalcium aluminosilicate.	*Dental World, Molfetta, Italy*
ADseal	Poly (1,4-butanediol) bis(4-aminobenzoate) (30–40 %), Bisphenol A diglycidyl ether–bisphenol A copolymer (20–30 %), 2-Hydroxyethyl salicylate (15–20 %), Triethanolamine (<10), calcium oxide (<5 %).	*Metabiomed, Cheongju, Korea*

a The chemical composition concentration data provided from the corresponding Material Safety Data Sheets (MSDS) and presented as a percentage by weight (WT%).

### 2.1 Material extracts

The following root canal sealers were used: ADseal (resin-based sealer) (Metabiomed, Cheongju, Korea), and the following bioceramic-based root canal sealers: VDW.1 seal bioceramic (VDW, München, Germany), and Fill Root ST (Dental World, Molfetta, Italy).

The root canal sealers were mixed under aseptic conditions inside a laminar flow hood, strictly following the manufacturers’ instructions. After setting, the samples were transferred to a culture medium (DMEM, Sigma-Aldrich Química SL, Madrid, Spain) and incubated at 37 °C in a humidified atmosphere containing 5 % CO_2_ for 24 h. Material extracts were then prepared according to ISO 10993-12 guidelines.^22^ As supported by previous studies, the extraction medium volume was determined based on a surface area-to-volume ratio of 1.5 cm^2^/mL.^23^ The resulting extracts were filtered for sterility and prepared in four concentrations: 100 % (undiluted), 50 % (1/2 dilution), 25 % (1/4 dilution), and 12.5 %.

### 2.2 Sample size calculation

The sample size calculation was based on a similar study.^24^ With an alpha level of 0.05 and a power of 84.7 %, a sample size per group of five test specimens was determined using G∗Power software version 3.1.9.7 (Heinrich Heine University Duesseldorf, Duesseldorf, Germany). The indicated samples were 10 for each group.

### 2.3 Cutler of cells

The human periodontal ligament stem cells (hPDLSCs) cell line (2 × 10^5^) was provided by the Egyptian company VACSERA. In culture flasks containing Eagle's minimal essential medium (MEM-E), cells were grown in a humidified atmosphere of 5 % CO_2_, 95 % air at 37 °C, and supplemented with 10 % fetal bovine serum (FBS), 1 % nonessential amino acid solution, 100 IU penicillin, and 100 units streptomycin in 0.9 % NaCl. (Sigma-Aldrich, Corp., St. Louis, MO, USA). For subsequent uses, target cells were kept in the lab. The growth medium was decanted, and trypsin (Sigma Aldrich, USA) solution (0.25 %) was added for two to 3 min. Following the decantation of trypsin, the cells were incubated until they completely dissociated. Following a hemocytometer count, the suspended cells were divided into 100 μl of culture media and arranged in 96-well tissue culture plates. To enable the cells to adhere, plates were incubated for 24–48 h at 37 °C in a 5 % CO_2_ environment.

### 2.4 Cell viability assay

Cytotoxicity was determined using a 3-(4,5-dimethylthiazol-2)-2,5-diphenyltetrazolium bromide (MTT) (Sigma-Aldrich, St. Louis, MO, USA) assay. The MTT test is used to assess the vitality of cells growing on the tested sealers. The sterilized specimens were transferred into a 96-well tissue culture plate and left there for a whole day at 37 °C with 5 % CO_2_. Subsequently, 100 μl/well (or 1 × 10^5^ cells/ml) are introduced to each specimen-containing well.

The specimens are incubated for one, three, and seven days, respectively, under the same circumstances. After being cleaned with phosphate-buffered saline (PBS), the samples are moved to different wells. Then, 200 μl of serum-free Dulbecco's Modified Eagle Medium (DMEM) containing 10 % fetal bovine serum (FBS) and 50 mg/ml of penicillin, streptomycin, and antifungal is combined with 100 μl of a 5 mg/ml solution of PBS. After that, the mixture is shaken for 5 min at 1500 rpm.^24^^,^^25^ While waiting for the MTT metabolism happens, the mixture is incubated for 5 h. After suctioning the leftover solution, 200 μl of Dimethyl sulfoxide (DMSO, Sigma-Aldrich Química SL, Madrid, Spain) is added to each well. The wells are then vigorously shaken at 150 revolutions per minute for 5 min, to ensure that the Formazan complex crystals are well dissolved. Optical density were determined using an ELISA reader (BioTek, Winooski, VT, USA) against a DMSO blank. All readings was done in triplicates at 570 nm.^24^^,^^25^ The data obtained for each group were reported as the mean and standard deviation.

The viability % and medication concentrations were plotted against each other. Optical density (OD) representing the% residual living cells was determined according to the following equation: Viability % = (OD test 1 × 100)/OD control.

Number of residual living cells = (OD of treated cells/OD of untreated cells) x Number of negative control cells.

### 2.5 Flow cytometry analysis

Using a fluorescence-activated cell sorting (FACSCAN) flow cytometer, flow cytometric DNA analysis was carried out.^26^ After treatment, the residual components of the cell pellets from both control and experimental samples were transferred into Falcon tubes. The cell culture medium was replaced with the binding buffer solution used for the cell cycle assay. Samples were centrifuged again at room temperature (20–25 °C) for 5 min at 300 rpm. To prevent disturbing the pellet at room temperature, the supernatant was aspirated, leaving about 50 μL of leftover buffer in the tubes. Next, 125 μL of solution “A" containing trypsin was added in each test tube and incubated for 10 min to allow lysis of cell membrane. Then, 125 μL of solution “B" which contains a trypsin inhibitor, was added to terminate the activity of trypsin in solution “A”. Subsequently, 125 μL of solution “C" was added. The tubes were then wrapped in aluminum foil to protect them from light and placed in an ice bath for at least 10 min. Samples were analyzed on the flow cytometer within 2–3 h. To asses apoptosis, Annexin-V and PI stains [Ex = 488 nm; Em = 530 nm] via the fluorescein isothiocyanate (FITC) signal detector (typically FL1) and PI staining by the phycoerythrin emission signal detector (typically FL2).

Based on Annexin V and PI staining, cells were categorized into four distinct populations: viable cells (Annexin V–/PI–), early apoptotic cells (Annexin V+/PI–), late apoptotic or secondary necrotic cells (Annexin V+/PI+), and necrotic cells (Annexin V–/PI+), as previously described.^27^

### 2.6 Remineralization potential

The preliminary remineralization potential for each root canal sealer was evaluated using optical emission spectroscopy (ICP-OES) (Ultima 2 ICP, Horiba, USA) to measure the release of calcium ions. To create discs with a 5 mm diameter and 2 mm thickness, each type of root canal sealer was placed into polytetrafluoroethylene tubes.^28^ After setting the sealer, every sample was immersed in a sealed flask holding 10 mL of distilled water at a starting pH of 7 and a temperature of 25 °C. The specimens were kept in an incubator at 37 °C and 95 % relative humidity. After 14 days, the total amounts of calcium ions released from each sealer were quantified (mg/L).^16^^,^^20^ A plastic syringe was used to remove 10 mL of each specimen's immersion solution, which was then pushed into a plastic falcon tube for ICP analysis with a wavelength range of 160–800 nm. Each falcon tube's sample solution was nebulized. After calibration, the intensity of the radiation emitted at the particular elemental frequency to detect the released calcium ions was analyzed to quantify the amount of each element present in solution.^28^, ^29^, ^30^

### 2.7 Statistical analysis

Exploration of the given data was performed using the Shapiro-Wilk test and the Kolmogorov-Smirnov test for normality; comparison between different groups was performed using the one-way ANOVA test, followed by Tukey's post-hoc test for multiple comparisons; and comparison between different intervals within the same group was performed using the repetitive one-way ANOVA. Data were conducted using SPSS 16.0 statistical software (SPSS Inc., Chicago, IL, USA). The significance level was set at P ≤ 0.05.

## 3 Results

### 3.1 Cell viability assay results

#### 3.1.1 Concentration 100 mg/ml

##### 3.1.1.1 Intergroup comparison

There was a significant difference among the groups after 24 h (fill root was significantly the highest, then VDW-1, while ADseal was significantly the lowest); after 72 h (VDW-1 was significantly the highest, then fill root, with an insignificant difference between them, while ADseal was significantly the lowest); and after 7 days, there was an insignificant difference between all groups. (VDW-1 was significantly the highest, then fill root, and ADseal with an insignificant difference between them were significantly the lowest), as represented in Table 2.

**Table 2:** The percentage of viable cells at concentrations of 100 mg/mL at different time intervals for all groups.

Sealers	24 h	72 h	7 days	P value
ADSeal	9.91 ^aA^ ± 1.64	9.39 ^aA^ ± 1.57	8.85 ^aA^ ± 1.11	0.86
VDW-1	26.13 ^aB^ ± 2.02	27.41 ^aB^ ± 5.48	20.75 ^bB^ ± 1.01	0.01∗
Fill Root.	30.43 ^aC^ ± 0.42	14.61 ^bC^ ±2.46	15.02 ^bC^ ± 3.50	0.0001∗
P-value	0.0001∗	0.0001∗	0.0001∗	

Means with different small letters in the same raw and those with different capital letters in the same column are significantly different∗ (P ≤ 0.05).

##### 3.1.1.2 Intragroup comparison

In ADseal, there was an insignificant difference between different intervals (P = 0.86); in VDW-1, there was a significant decrease in cell viability after 7 days (P = 0.01). In Fill Root, there was a significant decrease in cell viability% after 72 h, while there was an insignificant decrease after 7 days (P ≤ 0.05).

#### 3.1.2 Concentration 50 mg/ml

##### 3.1.2.1 Intergroup comparison

There was a significant difference between all groups after 24 h (P ≤ 0.05) as VDW-1 was significantly the highest, then Fill Root, with insignificantly the different, while ADseal was significantly the lowest); after 72 h, there was a significant difference among the groups as P ≤ 0.05 (VDW-1 was significantly the highest, then Fill Root, with insignificantly the different, while ADseal was significantly the lowest); and after 7 days, there was a significant difference between all groups as P ≤ 0.05. Fill Root and VDW-1 were significantly the highest, with insignificant differences between them, while ADseal was significantly the lowest, as represented in Table 3.

**Table 3:** The percentage of viable cells at concentrations of 50 mg/mL at different time intervals for all groups.

Sealers	24 h	72 h	7 days	P value
ADSeal	11.51 ^aA^ ± 0.56	10.63 ^aA^ ± 3.18	8.49 ^aA^ ± 2.53	0.06
VDW-1	72.14 ^aB^ ± 4.49	68.96 ^aB^ ± 6.66	41.03 ^bB^±3.76	0.0001∗
Fill Root	46.88 ^aC^ ± 5.52	36.33 ^bC^ ± 1.83	41.87 ^cB^ ± 4.19	0.0008∗
P-value	0.0001∗	0.0001∗	0.0001∗	

Means with different small letters in the same raw and those with different capital letters in the same column are significantly different∗ (P ≤ 0.05).

##### 3.1.2.2 Intragroup comparison

In ADseal, there was an insignificant difference between different intervals (P = 0.86); in VDW-1, there was an insignificant decrease after 72 h, while there was a significant decrease in cell viability% after 7 days (P ≤ 0.05). In Fill Root, there was a significant reduction in cell viability % after 72 h and after 7 days (P ≤ 0.05).

#### 3.1.3 Concentration 25 mg/ml

##### 3.1.3.1 Intergroup comparison

There was a significant difference between all groups after 24 h P ≤ 0.05 as (VDW-1 was significantly the highest, then Fill Root while ADseal was significantly the lowest), after 72 h there was a significant difference among the groups as P ≤ 0.05 (VDW-1 has the highest significant values with insignificant difference between them, followed by Fill Root while ADseal was significantly the lowest), after 7 days there was significant difference between all groups as P ≤ 0.05. (VDW-1 was significantly the highest, then fill root, while ADseal was significantly the lowest). As represented in Table 4.

**Table 4:** The percentage of viable cells at concentrations of 25 mg/mL at different time intervals for all groups.

Sealers	24 h	72 h	7 days	P value
ADSeal	12.76 ^aA^ ± 1.40	14.55 ^bA^ ± 1.03	10.72 ^aA^ ± 3.02	0.03∗
VDW-1	96.36 ^aB^ ± 2.33	94.79 ^aB^ ± 4.68	82.97 ^bB^ ± 1.23	0.0001∗
Fill Root	72.98 ^aC^ ± 3.43	54.21 ^bC^ ± 4.53	53.29 ^bC^ ± 1.95	0.0001∗
P-value	0.0001∗	0.0001∗	0.0001∗	

Means with different small letters in the same raw and those with different capital letters in the same column are significantly different∗ (P ≤ 0.05).

##### 3.1.3.2 Intragroup comparison

In ADseal, there were significant increases after 72 h, while there was a significant reduction after 7 days (P ≤ 0.05). In VDW-1, there was a significant reduction in cell viability after 7 days (P ≤ 0.05). In Fill Root, there was a significant reduction in cell viability after 72 h, while there was an insignificant reduction after 7 days.

#### 3.1.4 Concentration 12.5 mg/ml

##### 3.1.4.1 Intergroup comparison

There was a significant difference between all groups after 24 h P ≤ 0.05 as (VDW-1 has the highest significant values with an insignificant difference among them, then Fill Root while AD Sealer was significantly the lowest), after 72 h there was a significant difference among the groups as P ≤ 0.05 (VDW-1 has the highest significant values with an insignificant difference between them, then Fill Root while AD Sealer was significantly the lowest), and after 7 days there was a significant difference between all groups as P ≤ 0.05. (VDW-1 has the highest significant values; the fill root, ADseal, was significantly the lowest.) As represented in Table 5.

**Table 5:** The percentage of viable cells at concentrations of 12.5 mg/mL at different time intervals for all groups.

Sealers	24 h	72 h	7 days	P value
ADSeal	17.16 ^aA^ ± 0.90	14.11 ^abA^ ± 3.60	12.16 ^bA^ ± 1.32	0.01∗
VDW-1	99.49 ^aB^ ± 4.69	97.67 ^bB^ ± 5.38	90.37 ^cB^ ± 1.62	0.004∗
Fill Root	88.35 ^aC^ ± 2.46	62.36 ^bC^ ± 1.18	57.12 ^cC^ ± 3.41	0.0001∗
P-value	0.0001∗	0.0001∗	0.0001∗	

Means with different small letters in the same raw and those with different capital letters in the same column are significantly different∗ (P ≤ 0.05).

##### 3.1.4.2 Intragroup comparison

In ADseal, there was a significant decrease after 7 days; in VDW-1, there was a significant decrease in cell viability% after 72 h and after 7 days (P = 0.004). In Fill Root, there was a significant decrease in cell viability % after 72 h and after 7 days, as P ≤ 0.05.

### 3.2 Apoptotic effect results

The mean values of apoptosis and necrosis are listed in Table 6. The results revealed a significant difference between them, with Fill Root having the lowest values regarding total, early, late apoptosis, and necrosis, followed by VDW-1. ADseal sealer showed the significantly highest values regarding total, early, late apoptosis, and necrosis (P ≤ 0.05).

**Table 6:** The percentage of necrotic and apoptotic cells in the tested root canal sealers.

Sealers	Apoptosis			Necrosis
	Total	Early	Late	
AD Sealer	24.08^a^ ± 0.7224	7.13^a^ ± 0.2139	1.94^a^ ± 0.0492	15.31^a^ ± 0.4593
VDW-1	11.39 ^b^ ± 0.3417	2.16 ^b^ ± 0.0648	1.52 ^b^ ± 0.0516	7.51 ^b^ ± 0.2253
Fill Root	6.28^c^ ± 0.1884	0.71^c^ ± 0.0213	1.46^c^ ± 0.0438	4.11^c^ ± 0.1233
P value	0.0001∗	0.0001∗	0.0001∗	0.0001∗

Means with different small letters in the same raw and those with different capital letters in the same column are significantly different∗ (P ≤ 0.05).

### 3.3 Calcium ions release

The mean values of calcium ions emitted after 14 days of sealer storage are listed in Table 7. There was a significant difference in the values of the released calcium ions among the tested four sealers (P ≤ 0.05). The VDW-1 seal bioceramic showed the highest calcium ion release values, followed by Fill Root ST, while the ADseal root canal sealer showed the least calcium ion release values.

**Table 7:** The calcium ion release means and standard deviation (mg/l) for the different sealers.

Sealers	VDW.1 seal	Fill Root ST	ADseal sealer (control)	P-value
Calcium ions release	47.2^a^ ± 0.2	23.1 ^b^ ± 0.1	1.1^c^ ± 0.2	0.0001∗

Means with different letters in the same row show significant differences ∗ (P ≤ 0.05).

## 4 Discussion

Gutta-percha cones occupy most intra-canal spaces, with the remaining gaps being filled in with a small amount of root canal sealer. But by firmly closing the gap and shielding the tooth from bacterial infection, sealers function as a bridge between the gutta-percha and the tooth, allowing an endodontically treated tooth to survive.^31^

The purpose of this study was to evaluate three premixed, bioceramic-based root canal sealers with an epoxy resin-based root canal sealer in terms of their cytotoxicity, apoptotic effects, and mineralization potential using human periodontal ligament stem cell cultures. VDW-1 seal Bioceramic and Fill Root ST are bioactive in nature, i.e., when they come into contact with biological tissues, they promote mineralization.^32^ Since ADseal epoxy resin-based sealer is one of the most used and tested root-filling materials, it was utilized as a control.^33^^,^^34^ Moreover, ADSeal demonstrated superior biocompatibility compared to AH Plus, the gold standard of epoxy resin sealers; it was selected as the reference group rather than AH Plus.^31^^,^^35^

Because root canal sealers may come into close contact with periapical tissues during their setting and may be susceptible to blood flow and body fluids, their cytotoxicity is an important concern that needs to be considered.^36^ Sealers are intended to remain inside the root canal; however, they may be extruded into the apex through the lateral, auxiliary, and apical foramen inducing a detrimental biological consequence.^36^ Since sealers are chemically set and hardened mixes, their biocompatibility is negatively affected by the release of toxic substances during these reactions.^37^ The biological effects of root canal sealers have been assessed *in vitro* using a variety of techniques.^38^^,^^39^ The MTT assay is a standardized method utilized to test for cytotoxicity because of its ease of use, dependability, accuracy, simplicity, reproducibility, and time-saving features.^40^ The ISO 10993-5 guidelines state that sealers are cytotoxic if the percentage of viable cells is less than 70 %.^23^

The current study's findings indicate that the null hypothesis was rejected as there was a significant difference. A significant difference was exhibited among all four tested root canal sealers as regards cytotoxicity, apoptotic effects, and calcium ion release.

In this study, among different concentrations (100 %, 50 %, 25 %, and 12.5 %) and different evaluation time intervals (24 h, 72 h, and 7 days), VDW.1 seal bioceramic and Fill Root ST sealers showed higher cell viability than ADseal sealer. Furthermore, it was observed that as concentrations decreased, the values of the viability percentages increased.

ADseal sealer had the lowest mean percentage of viable cells in different evaluation periods in the current investigation. This may be attributes to presence leachable components of a residual monomer originated from incomplete polymerization, and liberation of heat during the polymerization reaction of the epoxy-based resin sealer.^41^^,^^42^

The observations made by Silva et al.^43^ and Torbati et al. ^44^ are in agreement with our results. They observed an increase in the cytotoxicity levels of epoxy resin-based sealer. This also agrees with the findings of Prati and Gandofli,^45^ and Eldeniz et al.,^46^ the results indicated that calcium silicate-based sealers have been demonstrated to elicit the proper biological responses than resin-based sealers. In consistent with our findings, other study revealed that the VDW-1Seal has showed a higher cytocompatibility in human periodontal ligaments cells.^47^. Previous research conducted by Seo et al.^48^ shown that sealers based on calcium silicate, exhibit more noticeable biological characteristics than sealers based on resin.

In this study, it was verified that Fill Root ST, followed by VDW.1 seal bioceramic demonstrated decrease cell apoptosis and necrosis; This result may be explained by the beneficial effects of the release of calcium ions from bioceramic-based sealers and their high solubility, thereby rendering them more alkaline. However, the cytotoxicity and increased cell apoptosis and necrosis of ADseal seen in this investigation is consistent with earlier findings.^49^ This finding may be caused by the presence of bisphenol A, which is known to be hazardous,^40^ as well as the formaldehyde released from amines used to speed up the polymerization of epoxy resin.^40^

This study demonstrates that calcium ions release of ADseal sealer was substantially lower than bioceramic-based root canal sealers: VDW-1 seal bioceramic, and Fill Root ST. This finding may be due to the chemical composition of the VDW-1 seal which is tricalcium and dicalcium silicate which many be contributed to the promotion of the hydration reaction.^50^

Bioceramic-based sealers showed a high cumulative calcium ions release which may be attributed to the presence of a different percentages of calcium silicates and calcium aluminates in their composition.^51^ In addition, bioceramic-based sealers characterized by high volume of open holes that create an internal network of water-filled pores, giving a more leaching of calcium ions.^52^ In particular, calcium ions release into the tissues may promote the remineralization potentiality by enhancement of calcium deposition, which promote bioactivity.^51^ These results coincided with a previous study that showed a significant rise in mineralized deposit development using calcium silicate-based sealers compared to epoxy resin-based sealers.^53^ Furthermore, according to a recent study using endodontic sealers conducted by López-García et al.^49^ showed that the Total Fill bioceramic-based sealer produced strong mineralization capacity than that of epoxy resin-based material.

However, despite the present *in vitro* study's simplicity, one of its limitations is that clinical conditions cannot always be precisely replicated in the laboratory. Variations in local pH, oxygen levels, and the patient's immunological condition could all have an impact on the results. Therefore, it is necessary to gather further confirmatory data, particularly to confirm that the clinical data agree with the laboratory results. Even though these findings are inspiring, more research is necessary to determine how they will affect periapical tissue repair. Additionally, more research is required to assess the microbiological penetration and examine the interface between root canal sealers and the dentinal walls.

## 5 Conclusions

As a whole, VDW.1 seal bioceramic and Fill Root ST sealers provide improved biocompatibility in terms of cell viability, apoptotic/necrotic effect, and remineralization potential. It should be emphasized that the biological characteristics of materials are strongly affected by their composition.

## Ethics approval and consent to participate

This *in vitro* study received ethical approval from the Medical Research Ethical Committee (MREC) of the National Research Centre (NRC), Cairo, Egypt (Reference number: 36572022). All methods were carried out in accordance with relevant guidelines and regulations. This study was carried out in accordance with the Declaration of Helsinki.

## Availability of data and materials

The data that support the findings of this study are available from the corresponding author upon reasonable request.

## Consent for publication

Not applicable.

## Patient's Guardian's consent

Not applicable.

## Funding

Not applicable.

## Declaration of competing interest

The authors declare that they have no known competing financial interests or personal relationships that could have appeared to influence the work reported in this paper.
